# Testicular, Spermatic Cord, and Scrotal Soft Tissue Sarcomas: Treatment Outcomes and Patterns of Failure

**DOI:** 10.1155/2021/8824301

**Published:** 2021-03-05

**Authors:** Varun K. Chowdhry, John M. Kane, Katy Wang, Daniel Joyce, Anne Grand'Maison, Gary N. Mann

**Affiliations:** ^1^Department of Radiation Medicine, Roswell Park Comprehensive Cancer Center, Buffalo, NY, USA; ^2^Department of Surgical Oncology, Roswell Park Comprehensive Cancer Center, Buffalo, NY, USA; ^3^Department of Biostatistics and Bioinformatic, Roswell Park Comprehensive Cancer Center, Buffalo, NY, USA; ^4^Department of Surgical Oncology, Cleveland Clinic, Cleveland, OH, USA; ^5^Department of Medicine, Roswell Park Comprehensive Cancer Center, Buffalo, NY, USA

## Abstract

**Introduction:**

Paratesticular sarcomas are defined as tumors that arise within the scrotum and include the subsites of epididymis, spermatic cord, and tunica vaginalis and represent the most common type of GU sarcoma. The mainstay of treatment is often surgical resection, combined with histology specific chemotherapy and radiotherapy. Due to the rare nature of the disease, there are limited data to guide management. We present our single-institution retrospective experience regarding the management and treatment of paratesticular sarcomas.

**Materials and Methods:**

We queried our oncology registry database for patients treated for testicular, spermatic cord, and scrotal soft tissue sarcomas between 1971 and 2017. Patients in this series had pathological confirmation of a sarcoma diagnosis by a sarcoma-specialized pathologist. Only patients with localized disease were included in this analysis with the exception of patients with a diagnosis of rhabdomyosarcoma where patients with both localized and metastatic disease were included on this study.

**Results:**

A total of 34 patients were included in this retrospective analysis. The median was 24 (range, 5–78), and the median tumor size was 6.25 cm. Twenty-six patients had localized disease (76.6%) at the time of diagnosis. A predominance of patients had tumors involving the spermatic cord (45.5%), and the most common histology was rhabdomyosarcoma (35.3%), leiomyosarcoma (26.5%), and well-differentiated liposarcoma (23.5%). The median follow-up was 71.0 months (range, 2.5–534.4 months). A total of 7 patients experienced an isolated local failure (20.6%), four patients developed distant metastatic disease (11.8%), and one patient (2.9%) with synovial sarcoma of the spermatic cord experienced a regional recurrence. The median progression-free survival (PFS) was 99.6 months, 95% CI (45.8–534.3 months), with a three-year PFS rate of 71%, 95% CI (53%–83%), and a 5-year PFS rate of 64% (range, 46%–78%). We did not find any statistically significant associations based on surgery type (*p*=0.15), the use of chemotherapy, (*p*=0.36), or final margin status (*p*=0.21). Two patients who were treated with preoperative radiotherapy had significant wound healing complication with chronic sinus tracts, though these patients did not experience a local recurrence.

**Conclusions:**

We provide a characterization of the natural history and treatment patterns of paratesticular sarcomas. While effective at reducing a local recurrence, preoperative radiotherapy was associated with significant toxicity. As a result, we prefer the use of postoperative radiotherapy in patients as clinically indicated. We did not find any specific treatment patterns associated with an improvement in clinical outcomes.

## 1. Introduction

Soft tissue sarcomas are an uncommon diagnostic entity, accounting for approximately 1% of all adult malignancies [1]. Paratesticular sarcomas account for an even rarer subset of soft tissue sarcomas, accounting for 1% of soft tissue sarcomas. However, paratesticular sarcomas are the most common presentation of genitourinary sarcomas, accounting for 30% of the overall cases [2].

Paratesticular sarcomas are defined as tumors that arise within the scrotum and include the epididymis, spermatic cord, and tunica vaginalis [3]. Patients will often present with scrotal swelling and a painless mass. Commonly, patients will undergo surgical resection without the clinical suspicion of sarcoma [4], and thus, consideration is given to additional surgery, chemotherapy, and radiotherapy. Much of the experience describing clinical patterns of behavior comes from small case series [5, 6] and have provided a framework to delineate pathways of treatment. Nevertheless, there is limited published experience on how to manage soft tissue sarcomas in this particular disease location. In general, principles of therapy from soft tissue sarcomas are employed, incorporating wide local resection, radiotherapy, and histologically driven systemic therapy as appropriate. However, the primary treatment modality for paratesticular sarcomas is considered to be surgical resection, with chemotherapy and radiotherapy utilized to facilitate surgical resection and decrease the local and distant recurrence [7]. Unlike the surgical approach for a primary testicular cancer which involves a radical inguinal orchiectomy with high dissection of the spermatic cord, surgery involves *en bloc* resection with negative margins.

In addition to oncologically oriented resection with negative margins, retroperitoneal lymph node dissection (RPLND) can be considered in patients either with retroperitoneal lymph node involvement or at having a high risk of nodal involvement. Here, we present our retrospective experience from a single institution regarding the management of testicular, spermatic cord, and scrotal soft tissue sarcomas.

## 2. Methods

After obtaining approval from our Institutional Review Board, our oncology registry database was retrospectively queried for patients treated for testicular, spermatic cord, and scrotal soft tissue sarcomas between 1971 and 2017. All patients in this series had pathological confirmation by a sarcoma-specialized pathologist either from a biopsy performed at our institution or secondary review of outside slides. For patients with rhabdomyosarcoma, patients with both localized and metastatic disease were included. For patients with other disease histologies, only patients with localized disease were included. Demographic, clinical, and pathological information was retrospectively recorded into a database. Survival curves were constructed using the Kaplan–Meier estimator method. Statistical Analysis was performed using SAS software (SAS v9.7; 100 SAS Campus Drive; Cary, NC 27513).

## 3. Results

A total of 34 patients were identified from our database. Demographic, clinical, and pathological information for patients in this series is shown in Table 1. The median age for patients in this series was 24 (range, 5–78), and the median tumor size was 6.25 cm. Twenty-six patients had localized disease (76.6%) at the time of diagnosis. Breakdown by tumor site and histology is reported in Table 1. A predominance of patients had tumors involving the spermatic cord (45.5%), and the most common histology was rhabdomyosarcoma (35.3%), leiomyosarcoma (26.5%), and well-differentiated liposarcoma (23.5%). Eighty-five percent of patients had a biopsy/nononcologic resection in order to make a sarcoma diagnosis, including 73.5% via a transinguinal incision approach. Slightly more than one-third of patients underwent a formal sarcoma wide resection with *en bloc* removal of the tumor and surrounding tissues. Treatment characteristics are shown in Table 2.

The median follow-up was 71.0 months (range, 2.5–534.4 months). A total of 7 patients experienced an isolated local failure (20.6%), four patients developed distant metastatic disease (11.8%), and one patient (2.9%) with synovial sarcoma of the spermatic cord experienced a regional recurrence in lymph nodes. No patient experienced a both a local and distant failure.

Of the seven patients who had local recurrences, we have detailed postrecurrence treatment information on four of those patients. Two of these patients had recurrence after an initial nononcological surgery, but had oncological re-excision and remained disease free. One patient had an asymptomatic local recurrence for a well-differentiated liposarcoma and chose no further therapy. The fourth patient who underwent reresection passed away during the immediate postoperative period.

**Figure 1:**
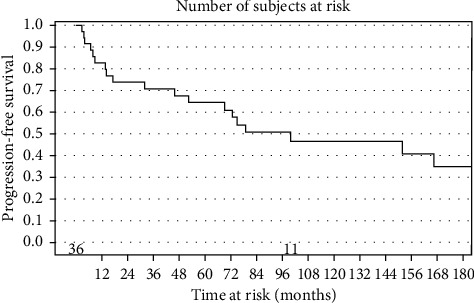
Kaplan–Meier curves for progression-free survival, all patients.

**Figure 2:**
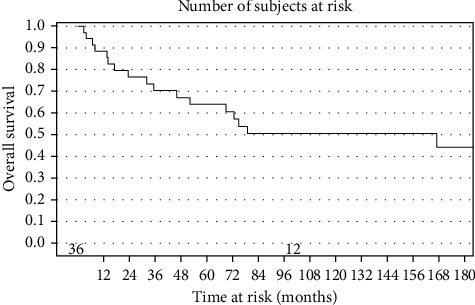
Kaplan–Meir curve for overall survival, all patients.

Survival curves for progression-free survival and overall survival are shown in Figures 1 and 2. For all patients in the cohort, the median progression-free survival (PFS) was 99.6 months, 95% CI (45.8–534.3 months), with a three-year PFS rate of 71%, 95% CI (53%–83%), and a 5-year PFS rate of 64% (range, 46%–78%). No local failures were noted in patients who received preoperative radiotherapy, but only two patients in this series were treated with preoperative radiation therapy for which follow-up data was available. However, both of these patients experienced significant wound healing issues with chronic sinus tracts.

**Figure 3:**
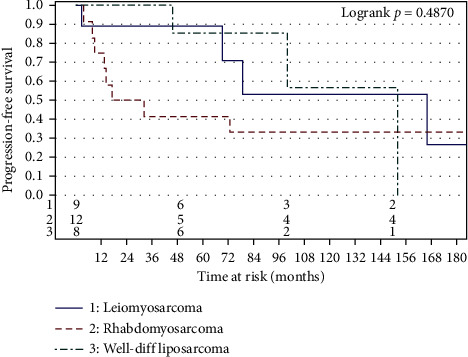
Progression-free survival by histology (for histologies, *n* ≥ 8).

**Figure 4:**
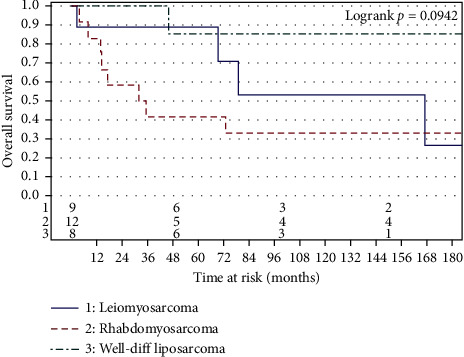
Overall free survival by histology (for histologies, *n* ≥ 8).

We had at least 8 patients for the following histological subtypes: leiomyosarcoma, Rhabdomyosarcoma, and well-differentiated liposarcoma. PFS and OS are shown for these histological subtypes in Figures 3 and 4, respectively. It is noteworthy that patients with well-differentiated liposarcoma had a 3-year progression-free survival of 100% and a five-year progression-free survival of 86%, 95% CI (33%–98%). On the other hand, patients with rhabdomyosarcoma had a three-year progression-free survival of 42%, 95% CI (15%–67%).

The median overall survival (OS) was 99.6 months, 95% CI (45.8–534.3 months), with a three-year OS rate of 71%, 95% CI (53%–83%) and a 5-year OS rate of 64% (range, 46%–78%). We did not find any statistically significant associations based on surgery type (*p*=0.15), the use of chemotherapy, (*p*=0.36), or final margin status (*p*=0.21).

## 4. Discussion

Adult paratesticular sarcomas represent a rare diagnostic entity, and often, initial management and surgical resection occur without clinical suspicion of sarcoma. Magnetic Resonance Imaging (MRI) can help delineate paratesticular tumors from tumors arising in the testis [8]. Core biopsy prior to undertaking definitive therapy can be useful, especially to guide appropriate preoperative therapy. Positron-emission tomography (PET/CT) has been shown to be beneficial to evaluate nodal and distant metastatic staging in patients with rhabdomyosarcoma [9].

The surgical treatment of paratesticular sarcomas is challenging secondary to the close proximity to the reproductive organs, the abdominal wall/inguinal canal, and even the free intra-abdominal cavity. As with other soft tissue sarcomas, negative margin wide resection is the potentially curative treatment. The wide resection field needs to include any prior surgical biopsy site secondary to the potential for microscopic tumor seeding. In a series of pediatric patients with paratesticular rhabdomyosarcoma, scrotal violation has been shown to result in inferior outcomes, unless adequately addressed [10]. The conventional urology teaching for the biopsy of a possible testicular cancer is radical orchiectomy via an inguinal approach to prevent tumor seeding of the scrotum with possible secondary drainage to the inguinal lymph nodes. However, this algorithm is suboptimal for paratesticular sarcomas as an inguinal orchiectomy approach mandates subsequent *en bloc* resection of the entire inguinal skin/canal with a complex abdominal wall reconstruction. The need for full-thickness abdominal wall/inguinal canal resection also potentially exposes the free intra-abdominal cavity to microscopic tumor cells. Consequently, if there is a high suspicion for a paratesticular sarcoma (as opposed to testicular cancer), either image-guided trans-scrotal core needle biopsies or surgical biopsy via a trans-scrotal approach is preferred. An *en bloc* hemiscrotectomy to include all tissues at risk for microscopic tumor is more technically straightforward than abdominal wall resection/reconstruction. Limiting the tumor to the hemiscrotum also results in a more defined target for either preoperative or postoperative radiation.

When wide resection was not technically possible secondary to the extent of microscopic tumor seeding or patient comorbidities, strong consideration was given to adjuvant radiation therapy to improve local control. In contrast, preoperative radiation was often pursued when wide resection was being considered, but close or microscopically positive resection margins were anticipated. A preoperative radiation approach is standard for most extremity and truncal soft tissue sarcomas as there are both theoretical and clinical advantages as compared to postoperative radiotherapy. The therapeutic efficacy of preoperative radiation therapy is enhanced as compared with postoperative radiation therapy due to tumor oxygenation allowing the same therapeutic effect to be achieved at a lower radiation dose [11]. Additionally, treatment volumes are larger in the postoperative setting and include more normal tissue as the tumor will displace normal structures out of the radiation field in the preoperative setting. From the extremity sarcoma literature, preoperative radiotherapy has been associated with an increased risk of acute, reversible toxicity but with the benefit regarding the reduction in late toxicity [12, 13]. Although neither of the patients who received preoperative radiation therapy experienced a local recurrence in our series, both patients developed postoperative wound healing issues with chronic sinus tracts. Therefore, our institutional preference for paratesticular sarcomas undergoing wide resection has actually changed over time to favor postoperative radiation once the surgical site has completely healed, especially given the potential morbidity of groin radiation due to skin folds, high bacterial counts, significant moisture, etc.

The use of neoadjuvant and adjuvant chemotherapy in sarcomas has always been a source of debate and remains controversial, given the lack of strong data. As a result, the patients in this series who received chemotherapy were largely patients with RMS and were treated with a vincristine-, adriamycin-, and cyclophosphamide-based regimens. Neoadjuvant chemotherapy has several advantages including downsizing the tumor, allowing less extensive surgical excision, and testing chemotherapy sensitivity but most importantly, treating micrometastases and potentially reducing the risk of developing metastatic disease. No randomized placebo-controlled trial has yet confirmed a survival benefit from neoadjuvant chemotherapy. Nevertheless, many centers are using preoperative chemotherapy in patients at high risk of developing metastatic disease based on sarcoma histopathology, tumor size, and grade. The use of neoadjuvant chemotherapy in sarcoma remains institution dependent. With the exception of rhabdomyosarcoma, neoadjuvant chemotherapy was not largely used during the period this cohort of patients was treated, therefore limiting any conclusion about the efficacy of neoadjuvant chemotherapy in paratesticular sarcomas. The role of adjuvant chemotherapy in sarcoma has been studied more extensively. The strongest evidence of survival benefit comes from the meta-analysis published by Pervaiz et al. [14] who demonstrated a 11% absolute risk reduction of death. Since only 11.7% of patients in our cohort received adjuvant chemotherapy, our data are insufficient to draw a meaningful conclusion about the benefit of adjuvant chemotherapy in paratesticular sarcomas.

We will now address some of the limitations of our paper. As a retrospective analysis, it is difficult to make any definitive conclusions regarding treatment pathways that could be best for patients. As a tertiary referral center, many patients came from the surrounding community, and often, we only have follow-up data regarding surgical outcomes, while other variables, including chemotherapy and radiotherapy details, are missing. Additionally, given the small number of patients, combined with several different histopathologies, we did not find any specific disease- or treatment-related factors that were definitively associated with improved outcomes. It is also important to note that while as a practice, we centrally review of pathology by a sarcoma-trained histopathologist, we did have limited ability to capture more specific histopathological information for patients included from earlier time points of the database. For example, we were unable to capture specific types of rhabdomyosarcoma for some patients as a result.

It is noteworthy that our calculated progression-free survival and overall survival are nearly identical, as for many patients, the most recent follow-up we have is the date of death. We found a median overall survival (OS) of 99.6 months, 95% CI (45.8–534.3 months), with a three-year OS rate of 71% and a 5-year OS rate of 64%. Our results are consistent with the experience at the Memorial Sloan Kettering Cancer Center, who reported a five-year disease-free survival of 75% [15].

We did not demonstrate differences in clinical outcome by surgery type. Although we believe in the importance in *en bloc* excision for selected patients, there are several variables that can confound the importance of surgical excision with widely negative margins. With the exception of patients with well-differentiated liposarcomas, patients with scrotal sarcomas are at high risk of both local and distant recurrence. Therefore, the potential benefits of radiation and systemic chemotherapy must be reconciled with the limited benefits of an extensive, technically complex, morbid wide resection that could otherwise delay systemic therapy. Furthermore, the number of patients in this series may be too small to stratify by other important variables such as histology, tumor size, and grade. Additionally, patients were offered treatment based on pretreatment and disease-specific associated risk factors. As this is an uncontrolled series, the diverse histologies likely impacted the treatment a patient would have received. For example, a patient with a small well-differentiated liposarcoma may have a favorable disease course even without further treatment. On the other hand, a patient with large, high-grade tumor was offered more aggressive therapies, making it difficult to analyze the efficacy of the particular therapy. Additionally, over one-third of the patients in this series had a diagnosis of a rhabdomyosarcoma, where the mainstay of treatment involves systemic chemotherapy and radiotherapy. Therefore, aggressive surgical resection may provide little added benefit due to the high rates of local control that can be achieved with other therapies.

The role of RPLND in the management of scrotal and paratesticular soft tissue sarcomas is considered controversial [16]. Catton et al. reported that patients presenting with primary disease had a regional recurrence rate of 28% suggesting that patients with rhabdomyosarcoma, fibrous histiocytoma, or fibrosarcoma may require RPLND [17]. Our data suggest RPLND is not needed for most patients with paratesticular sarcomas. We only had one patient (2.9%) with an isolated regional failure in the inguinal lymph nodes in a patient diagnosed with synovial sarcoma on the spermatic cord. Regional nodal recurrence rates are uncommon in extremity soft tissue sarcomas [18], and our data also suggest that regional lymph node recurrence of paratesticular soft tissue sarcomas is rare as well. The low rate of nodal relapse in our series is consistent with other published reports [19]. As a result, our data suggest that routine lymph node dissection and radiotherapy to regional lymphatics is not indicated for most paratesticular soft tissue sarcomas. The exception of this is patients with paratesticular rhabdomyosarcoma benefit from RPLND, where the available data suggest that imaging incorrectly stages less than half of the patients, and thus, surgical ipsilateral retroperitonal lymph node dissection is considered to be the standard of care [20].

## 5. Conclusions

We describe our treatment approach and philosophy regarding the management of paratesticular and scrotal soft tissue sarcomas, as well as outcomes of patients treated at our institution. Although our series does not definitely demonstrate the importance of oncological surgery with widely negative margins, we agree with the guidelines from the National Comprehensive Cancer Network (NCCN) that obtaining oncologically appropriate margins is important for most patients, with consideration of radiation therapy and chemotherapy in selected patients based on specific type [21]. Due to significant toxicity experienced in patients treated preoperative radiation therapy, we favor postoperative radiation therapy once all surgical wounds have healed. Finally, neoadjuvant chemotherapy should be considered in high-grade histology to reduce the risk of developing metastatic disease and improve outcome based on pathologically driven treatment paradigms for sarcomas that occur in different anatomic subsites.

## Figures and Tables

**Table 1:** Demographic information.

Total number of patients (*N*)	34
Median age at diagnosis (years)	42 (range, 5–81)
Median tumor size (cm)	6.25 (range, 0.8–14)

*M stage at diagnosis*	
M0	26 (76.6)
M1	7 (20.6%)
Unknown	1 (2.9%)

*Tumor site*	
Scrotal	11 (33.3%)
Spermatic cord	15 (45.5%)
Testis	8 (21.2%)

*Histology*	
Well-differentiated liposarcoma	8 (23.5%)
Dedifferentiated liposarcoma	1 (2.9%)
Rhabdomyosarcoma	12 (35.3%)
Embryonal rhabdomyosarcoma	3 (25%)
Unknown type	9 (75%)
Leiomyosarcoma	9 (26.5%)
Myxoid liposarcoma	2 (5.9%)
Synovial sarcoma	2 (5.9%)

*Method of diagnosis*	
Excisional biopsy/transcrotal surgery	14 (41.2%)
Inguinal orchiectomy	18 (52.9%)
Hernia repair	1 (2.9%)
Transcrotal surgery	7 (20.6%)
No biopsy, definitive surgery only	1 (2.9%)

*Biopsy/nononcological resection prior to surgery*	
Yes	29 (85.3%)
No	4 (11.8%)
Unknown	1 (2.9%)

**Table 2:** Treatment.

*Type of definitive surgery*	
*Local surgery*	
No local surgery^*∗*^	12 (35.3%)
WLE, en bloc removal	13 (38.2%)
Inguinal orchiectomy	7 (20.6%)
Missing	2 (5.9%)

*Nodal dissection*	
Retroperitoneal node dissection	5 (14.7%)

*Margin status*	
Positive	3 (8.8%)
Negative	11 (34.4%)
Close (<1 mm)	2 (5.9%)
Unknown margin status	18 (52.9%)

*Chemotherapy*	
Adjuvant	4 (11.7%)
Therapeutic	6 (17.6%)

*Radiotherapy* ^*∗∗*^	
Any radiation therapy	7 (34%)
Preoperative	3 (8.8%)
Postoperative	4 (11.8)
Mean preoperative radiation dose^*∗∗∗*^	5220 cGy (range, 5000–5040 cGy)
Whole abdomen radiation dose (RMS only)	1805 cGy

^*∗*^Some patients with no local surgery had nononcological WLE for diagnostic purposes prior to presentation to the tertiary center. ^*∗∗*^ Patients who were known to have radiation therapy at our facility. ^*∗∗∗*^All patients in this series who were treated with postop radiotherapy were treated at another facility, and further treatment records regarding dose are not available.

## Data Availability

The data used to support the findings of this study are available from the corresponding author upon request.
